# A Royal Hospital Sunday

**Published:** 1903-06-13

**Authors:** 


### HOSPITAL ADMINISTRATION.

### CONSTRUCTION AND ECONOMICS.

### A ROYAL HOSPITAL SUNDAY.

#### THE KING AND QUEEN AT ST. PAUL'S CATHEDRAL.

For the first time on record the Sovereign of these realms publicly visited St. Paul's on Sunday, 7th inst., for another purpose than that of thanksgiving for recovery from illness or victory over the nation's foes. The event thus becomes historic, and the circumstances attending it of unique and national concern. When it was announced that King Edward and Queen Alexandra would attend the afternoon service in the Cathedral in connection with Hospital Sunday, it was at once recognised that the occasion would illustrate in a remarkable manner the interest personally taken by their Majesties in the welfare of their sick and suffering subjects. It is true that no evidence of this fact, which has so repeatedly been demonstrated, was needed; but the presence of the Sovereign and his Consort at a service intended as a prelude to the services on Hospital Sunday, June 14th, could not, it was felt, fail to give a valuable impetus to the movement and to accentuate the importance of the claims of the great medical charities of the metropolis.

The Congregation.

How eagerly people of all classes availed themselves of the opportunity of joining with their Majesties in a solemn act of worship was shown some time before the appointed hour arrived. Long ere the Cathedral doors were opened at two o'clock a large crowd had assembled, and that portion of the building which was free to all was filled in a few minutes. Nor did the holders of tickets, who were bidden to be in their places by 2.45, take advantage of the time limit. The majority preferred to be in possession of their seats soon after two, and, thanks to the excellent arrangements supervised by Sir Edward Hay Currie, Secretary of the Hospital Sunday Fund, and carried out by his son, with several courteous assistants, there was no difficulty in reaching them. It was easy to see that the congregation was of a thoroughly representative character, and included, as was desired, not only distinguished but humble individuals. Most prominent, as a matter of course, was the Lord Mayor in his robes and chain of office. The scarlet of the tunics of the Sheriffs and the gowns of the Aldermen, the Mazarin-blue of the gowns of the Common Councillors, an occasional uniform, and here and there the brilliance of a lady's costume, gave a picturesque touch to the scene. But the pervading element was that of unobtrusiveness. People had obviously not met together to show themselves off, as at a fashionable function, but in the spirit of simplicity and reverence which was strictly in keeping with the nature of the service. The neat but unassuming costumes of the long line of nurses from all the leading hospitals who flanked the passage down the middle of the nave, and in effect formed a guard of honour, or were accommodated with seats in the chancel, harmonised admirably with the surroundings, while they also afforded an object-lesson of the reality of the work which the congregation were subsequently invited to support. They had come direct from "the mansions of sorrow and pain"—which Howard, whose statue adorns the Cathedral, visited in the philanthropic tour that secured him undying fame—for the purpose of joining in the prayer for the Divine blessing, "especially for all those in the London hospitals."

Arrival of the Royal Party.

The venerable Duke of Cambridge, accompanied by his son, Colonel FitzGeorge, was the first member of the Royal Family to take his seat in the space set apart for their use beneath the dome facing the altar rails; and then in quick succession arrived the Duke of Connaught and his two daughters, the Princesses Margaret and Patricia—who were later joined by Princess Victoria—Princess Henry of Battenberg, Princess Victoria Eugénie, Princess Christian, the Princesses Victoria and Louise Augusta of Schleswig-Holstein, and finally by the Prince and Princess of Wales. Just at the moment that the rays of the summer sun were wanted to brighten the scene the authorities turned on the electric light, and simultaneously the strains of the National Anthem outside and of the magnificent organ within were the signal that the King and Queen had arrived. The procession, headed by the cross-bearer, slowly marched up the nave, and consisted of the Canons Residentiary, the Remembrancer, the Sheriffs, the Lord Mayor, his Majesty the King, supported by the Bishop of London, and her Majesty the Queen, supported by the Dean of St. Paul's. At this stage the choir and clergy proceeded from the vestry to the chancel. The King and Queen having taken their seats the hymn "Bright the vision that delighted" was sung.

The Service.

The service was as stately as it was simple. Except that two special Lessons were selected, as well as read, by the Dean, and that the General Thanksgiving was omitted, there was no departure from the Evensong appointed for Trinity Sunday. After the third Collect, Stainer's anthem, "I saw the Lord sitting upon a Throne, high and lifted up," was rendered with fine effect, and before the sermon "Thine arm, O Lord, in days of old" was sung. The preacher was the Bishop of Stepney, canon in residence, who took his text from St. Luke xxii. verses 24, 25, 26:—"And there was also a strife among them, which of them should be accounted the greatest. And He said unto them, The Kings of the Gentiles exercise lordship over them; and they that exercise authority upon them are called benefactors. But ye shall not be so: but he that is greatest among you, let him be as the younger; and he that is chief, as he that doth serve."

The Sermon.

Dr. Lang, whose discourse occupied less than a quarter of an hour in delivery, spoke with great clearness and emphasis, and must have been well heard at some distance from the pulpit. When he had dealt in a general sense with the question raised in the text, and eloquently enforced the lesson that the true royalty of human life is its capacity of compassion, he reminded his vast congregation that that afternoon the appeal for their compassion was one of peculiar urgency. Hospital Sunday brought before their imagination the vast tide of pain and suffering which the hospitals were trying with such noble patience and devotion to stem. If a single poor sufferer were brought before them, there was not one who would not do the best that was possible to help. But that day it was not one poor sufferer, but the 105,000 in-patients and the 1,670,000 out-patients in the London hospitals who were laid at their door. To add to the force of their plea our gracious King and Queen, with the members of the Royal House, had associated themselves with it by their presence, illustrating the true royalty of compassion, proving once again, though it needed no proof, that here in England "the Throne is upholden by mercy." Such an appeal on behalf of the poor and sick must act as a test of the real truth of our character. If it left us unmoved, then the poverty of life was discovered; it was plain that the disease of selfishness was upon us. On the other hand, if our hearts moved forth to meet the appeal in the ardour of compassion, there was still, in spite of all our sins and shortcomings, some element of greatness left in us—something God-given and God-like. What might not be done to establish on a sure foundation these homes of mercy, to banish the shadow of anxiety which haunted the efforts of the devoted men who were responsible for them, if every person in that great congregation asked, in eagerness for the answer, "What can I do, by thought and influence and self-denial, for the hospitals of London?"

"Lest We Forget."

The Bishop went on to point out that the hospitals stood in our midst as a constant reminder, "Lest we forget, lest we forget" the true greatness of the City of London—St. Bartholomew's, witnessing in the very centre of the City to the kindness of an olden time; the London Hospital, rising above the crowded streets of the East End; St. Thomas's, confronting the centre of the Empire's labours; St. George's, standing in mute appeal as the stream of wealth and luxury flows around it. The hospitals were voices calling to London, "Remember, O remember, the Lord thy God; remember the trust with which He has charged thee; remember the poor, the suffering, and the stranger at thy gates." There were many signs, especially the King's Fund, the generosity of men such as he who had again come forward to stimulate his fellow-citizens, the steady growth of the Hospital Sunday Fund, that the City is not deaf to the call. But could it be said that the wealth of London had risen to the height of its opportunity when the annual deficit of the hospitals was £250,000? There were ominous declarations that the strain of raising funds had reached the limit of endurance, and that ere long the hospitals must turn to the compulsory help of the rates. But good which was done under the compulsion of "must" could never have the moral worth of good which was done under the obligation of "ought." To remove from the walls of our hospitals the old familiar words, "Supported by voluntary contributions," would be an act of moral retreat on the part of a great and wealthy city. God grant that this notable day might mark the beginning of a more resolute and sustained effort by the great City of London to prove that its power, like the power of God, from whom it came and to whom it was responsible, is declared most chiefly in showing mercy and pity.

The Close.

The sermon over, the hymn "Lord of Glory, Who hast bought us" was sung, and the collection for the Hospital Sunday Fund was taken. About fifty gentlemen held the offertory bags, Mr. Richard B. Martin, M.P., handing one of white and gold to the King and Queen for their contribution. The alms having been deposited on the altar, the Blessing was pronounced by the Lord Bishop of London. The offertory amounted to £4,300.

